# Activation of Ca^2+^-sensing receptor as a protective pathway to reduce Cadmium-induced cytotoxicity in renal proximal tubular cells

**DOI:** 10.1038/s41598-018-19327-9

**Published:** 2018-01-18

**Authors:** Jie Gu, Shuya Dai, Yanmin Liu, Haitao Liu, Yao Zhang, Xingqi Ji, Feng Yu, Yang Zhou, Liang Chen, William Ka Fai Tse, Chris Kong Chu Wong, Binghai Chen, Haifeng Shi

**Affiliations:** 1 Institute of Life Science, Jiangsu University, Zhenjiang, Jiangsu 212000 China; 2 Medical Section, The Third Affiliated Hospital of Jiangsu University, Zhenjiang, Jiangsu 212000 China; 3 Faculty of Agriculture, Kyushu University, Fukuoka, Japan; 4 Department of Biology, Hong Kong Baptist University, Kowloon Tong, Hong Kong China; 5 Department of urology, Affiliated Hospital of Jiangsu University, Zhenjiang, Jiangsu 212000 China

## Abstract

Cadmium (Cd), as an extremely toxic metal could accumulate in kidney and induce renal injury. Previous studies have proved that Cd impact on renal cell proliferation, autophagy and apoptosis, but the detoxification drugs and the functional mechanism are still in study. In this study, we used mouse renal tubular epithelial cells (mRTECs) to clarify Cd-induced toxicity and signaling pathways. Moreover, we proposed to elucidate the prevent effect of activation of Ca^2+^ sensing receptor (CaSR) by Calcimimetic (R-467) on Cd-induced cytotoxicity and underlying mechanisms. Cd induced intracellular Ca^2+^ elevation through phospholipase C-inositol 1, 4, 5-trisphosphate (PLC) followed stimulating p38 mitogen-activated protein kinases (MAPK) activation and suppressing extracellular signal-regulated kinase (ERK) activation, which leaded to increase apoptotic cell death and inhibit cell proliferation. Cd induced p38 activation also contribute to autophagic flux inhibition that aggravated Cd induced apoptosis. R-467 reinstated Cd-induced elevation of intracellular Ca^2+^ and apoptosis, and it also increased cell proliferation and restored autophagic flux by switching p38 to ERK pathway. The identification of the activation of CaSR-mediated protective pathway in renal cells sheds light on a possible cellular protective mechanism against Cd-induced kidney injury.

## Introduction

Occupational and environmental pollutant of Cadmium (Cd) caused various organs damage, especially the kidney, which is the major site of Cd accumulation^1–3^. In kidney, the renal proximal tubule is the first opportunistic site of Cd reabsorption following plasma filtration in the glomerulus^4,5^. Therefore, the renal proximal tubular cells are excellent model to study Cd-induced cytotoxicity and renoprotective strategies^6,7^. Exposure to Cd could induce various cellular responses such as carcinogenesis, necrosis, apoptosis, proliferation and autophagy^8–10^. Previous studies had reported that Cd induced apoptotic cell death in the renal proximal tubule cells, i.e. porcine (LLC-PK_1_)^11^ and human (HK-2) proximal tubular epithelial cell^12^. More importantly, the molecular mechanisms underlying Cd-induced proximal tubular damage and renoprotective strategies are still in study.

Intracellular calcium homeostasis is very important in the control of many cellular processes^13–15^. Previous studies suggested that Cd disrupted intracellular Ca^2+^ homeostasis, resulting in cell apoptosis in a variety of cells^9,16–20^, including renal tubular cells^21,22^. Cd disrupted intracellular Ca^2+^ homeostasis through reducing the influx of extracellular Ca^2+^^23,24^, or increasing Ca^2+^ release from intracellular Ca^2+^ store^22,25^. Endoplasmic reticulum (ER) is a major intracellular store of Ca^2+^^26^ and Cd induces Ca^2+^ release from ER store, associated with ER stress through cation-sensing receptor (CSR) mediated phospholipase C (PLC)-inositol 1, 4, 5-trisphosphate (IP_3_) signaling pathway^18,27^. Cd induced elevation of intracellular Ca^2+^ level also triggers mitochondrial damage^18^, evoking reactive oxygen species (ROS) generation from mitochondria^19,22,28–30^. Both ER stress and mitochondrial damage lead to up-regulation of expression of caspase-3, resulting cell apoptotic death^16–18^. Additionally, intracellular Ca^2+^ signaling pathway also mediated Cd-induced autophagy^17^, which played a renoprotective role in both acute kidney injury and chronic kidney diseases^31^, and was indicated as a protective way against Cd-induced apoptosis in lung epithelial fibroblast cells WI38^32^, pheochromocytoma cell line PC-12^33^, and rat renal tubular cells^34^. However, initial autophagic protection would switch to disruption of autophagic flux and result in cell death during Cd stress accrual in renal NRK-52E cells^6^. Therefore, it is important to understand the roles of intracellular Ca^2+^ signaling pathways in Cd-induced apoptosis and autophagy, and their relationship in renal tubular cells.

In addition, a great number of studies have show that Cd regulates the functions of many Ca^2+^-dependent regulatory proteins such as protein kinase C (PKC), mitogen-activated protein kinase (MAPK), calmodulin (CaM), and calcium/calmodulin-dependent protein kinase II (CaMKII), inducing dysregulation of intracellular Ca^2+^ homeostasis^16,35–41^. Moreover, these intracellular signals can be induced by the extracellular calcium-sensing receptor (CaSR), a G-protein-coupled receptor (GPCR), which is responsible for the control of calcium homeostasis in body fluids^42–46^. Faurskov and Bjerregaard’s study showed the CaSR agonist, neomycin diminished Cd-evoked increase of intracellular Ca^2+^ in renal distal epithelial A6 cells^27^. However, the underlying mechanism and function of activation of CaSR on Cd-induced disruption of intracellular Ca^2+^ homeostasis and Cd-regulated pathways were still undeclared. In addition, although due to CaSR agonist neomycin and Gd^3+^ (Gadolinium ion) could not stimulate CSR, suggesting CaSR is different from CSR, both receptors mediate activation of PLC-IP_3_ pathway and intracellular Ca^2+^ level^27^. However, it is still unknown whether there is competition or crosstalk between CaSR and CSR mediated pathways.

The results of RT-PCR and immunohistochemistry staining had detected the expression of CaSR in rat renal proximal tubule^47–49^. Interestingly, our previous study indicated that activation of CaSR by calcimimetic R-467 could as a protective pathway to reduce Ca^2+^-induced cytotoxicity in gill cells of Japanese eels^50^. Given this observation together with previous reports on biological functions of CaSR, we attempt to decipher the protective role and its underlying mechanism in activation of CaSR against Cd induce cytotoxicity in renal proximal tubular cells. Here, we demonstrated that activation of CaSR by calcimimetic R-467 reduced Cd-evoked intracellular Ca^2+^ elevation, followed ROS generation and apoptosis, and R-467 also restored autophagic flux and increased cell proliferation by switching Cd-activated calcium-p38 MAPK to R-467 activated PLC-ERK pathway. Our findings provide the groundwork for future studies on renoprotective therapy of Cd-induced kidney injury.

## Results

### Detection of expressions of CaSR in renal cells and R-467 prevented Cd-induced renal cells death and cytotoxicity

**Figure 1:**
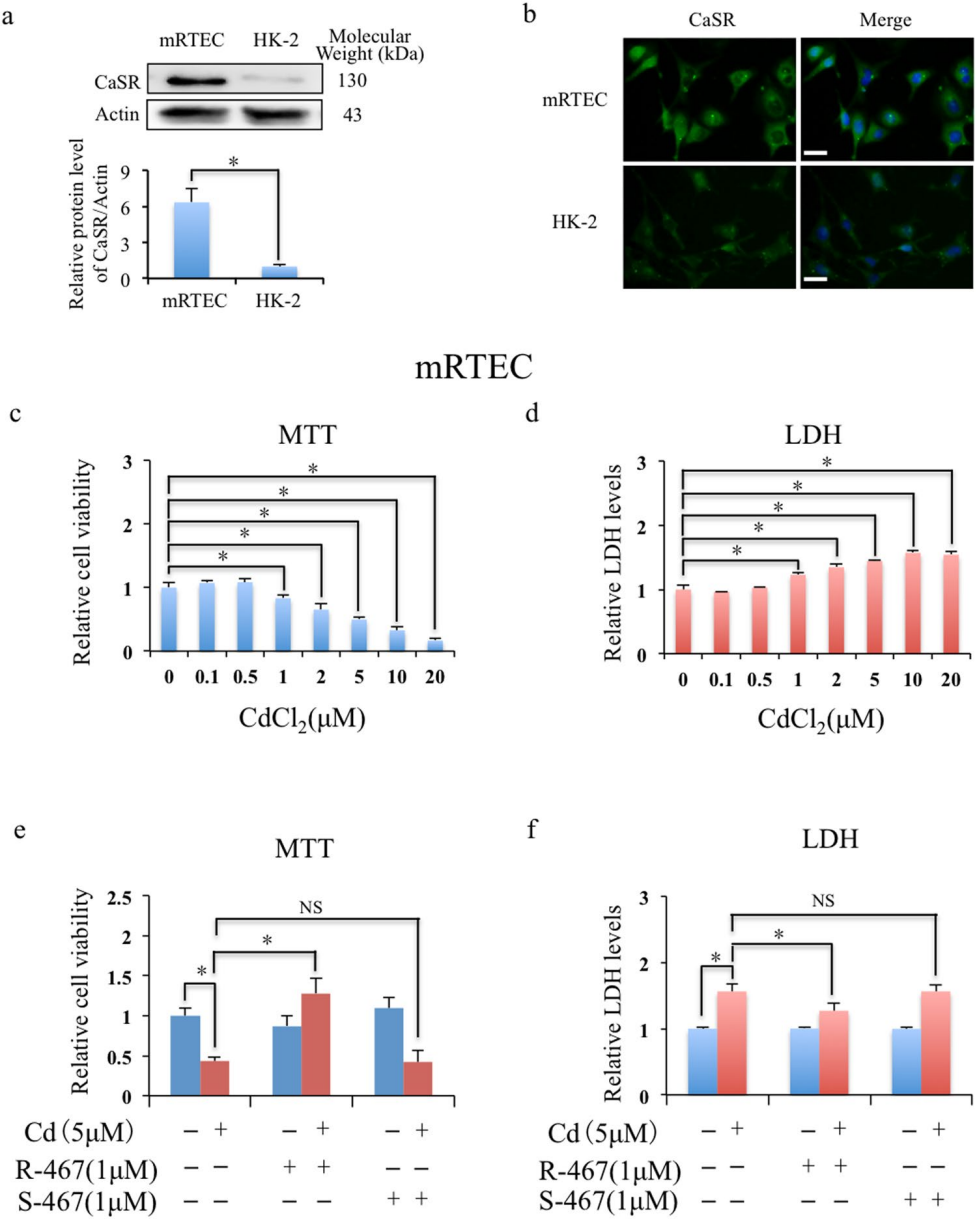
Expression of CaSR in renal cells and Cd induced cell death and cytotoxicity in mRTEC cells. (**a**) Western blotting shows expressions of CaSR in mRTEC and HK-2 cells. *P < 0.05, using Student’s t-test. (**b**) Immunofluorescence detection of expressions of CaSR in mRTEC and HK-2 cells. The cells were counterstained with CaSR (green) and DAPI fluorescence (blue). Scale bar: 20 μM. (**c,d**) Cd induced cytotoxicity in mRTEC. After treated with 0–20 μM Cd for 24 h, cell viability of mRTEC was evaluated by MTT assay. The condition mediums were collected for LDH cytotoxicity assay. Results are presented as mean ± SD (n = 4). (**e**,**f**) Effects of calcimimetics on Cd induced cytotoxicity in mRTEC. After treated with Cd (5 μM), R-467 (1 μM), S-467 (1 μM), Cd (5 μM) + R-467 (1 μM), or Cd (5 μM) + S-467 (1 μM) for 24 h, cell viability of mRTEC was evaluated by MTT assay. The condition mediums were collected for LDH cytotoxicity assay. Results are presented as mean ± SD (n = 4). *Statistical significance between control and treatments, or Cd treatment and co-treatment of Cd + R-467, *P < 0.05, using Student’s t-test.

To detect the expressions of CaSR in renal cells (i.e. mRTEC and HK-2 cells), western blotting (Fig. 1a) and immunofluorescence (Fig. 1b) were performed and the results showed higher expression of CaSR in mRTECs than HK-2 cells. The results of MTT assay (Fig. 1c) and LDH cytotoxicity assay (Fig. 1d) showed Cd induced cytotoxicity of renal cell mRTEC in a dose dependent manner from 1 μM, and LD50 (lethal dose, 50%) was around 5 μM that was used in the following experiments. However, Cd induced cytotoxicity were significantly prevented when co-treated with calcimimetic R-467, but not with S-467 in mRTEC cells (Fig. 1e,f). And consistent results were also detected in HK-2 cells (Fig. S1a–d). As a positive allosteric modulator of CaSR, calcimimetic R-467 (R-enantiomer) could enhance the sensitivity of activation of CaSR, but S-467 (S-enantiomer) is less activator. In hence, co-treatment of R-467 but not S-467 prevented Cd-induced cytotoxicity (Fig. 1e,f and Fig. S1c,d) and suggested that the protective effect was specific and effectively mediated by CaSR. It is noted that Cd could not affect the expressions of CaSR in mRTEC and HK-2 cells (Fig. S1e). Considering higher expression of CaSR in mRTEC cells, we chose mRTEC as cell model in the following experiments.

### R-467 increased PLC activity and reinstated Cd-induced intracellular Ca^2+^ level elevation

**Figure 2:**
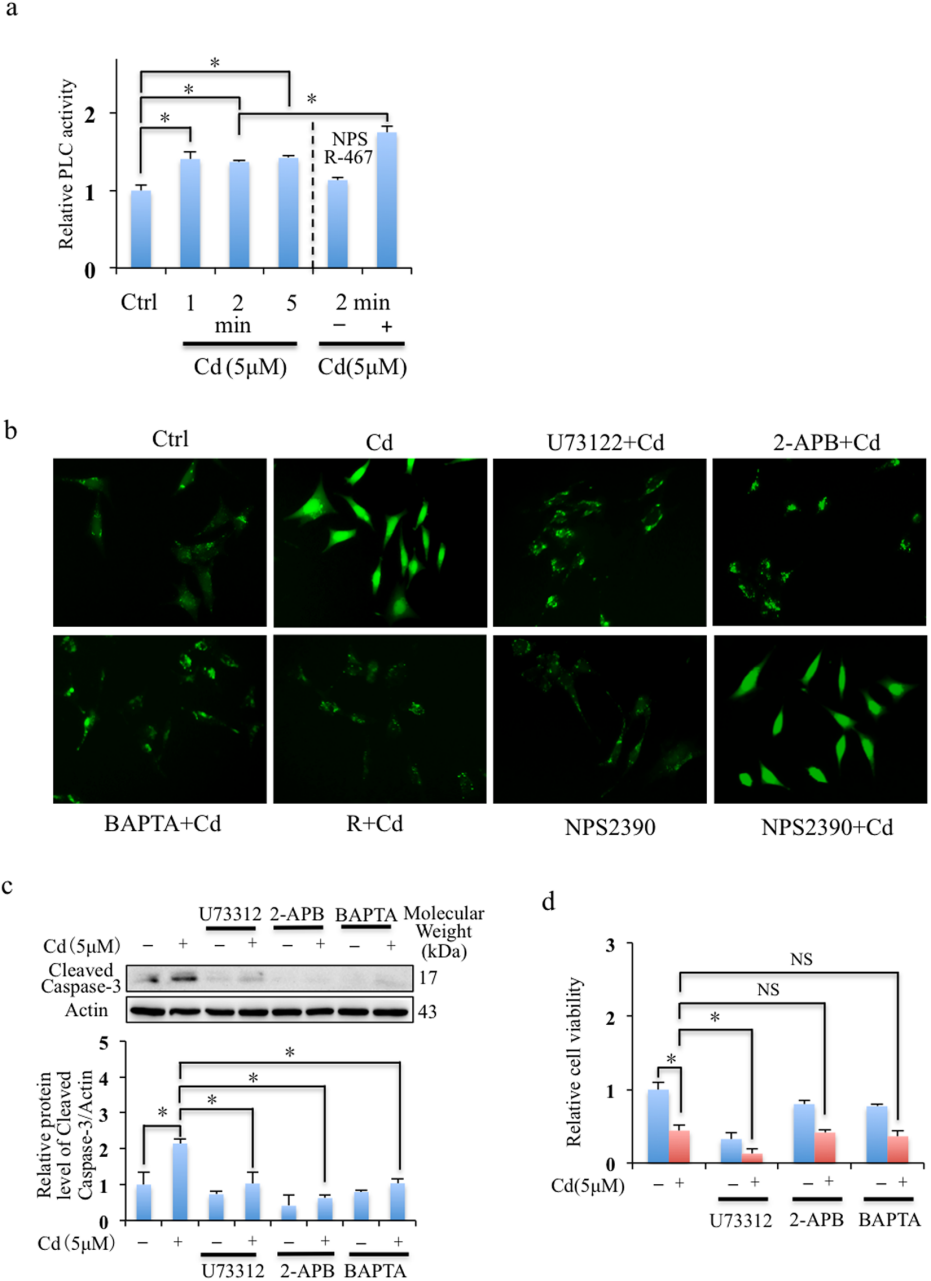
Effects of calcimimetics on Cd induced PLC activity and intracellular Ca^2+^ level elevation in mRTEC cells. (**a**) Effects of Cd and R-467 on PC-PLC activity in mRTEC cells. After post-exposed to Cd treatment (5 μM) for 1, 2, 5 min or co-treated with R-467 (1 μM) for 2 min, the PC-PLC activity of mRTEC cells were detected. Cd induced the activity of PC-PLC in mRTEC cells in a time-dependent manner. R-467 increased PC-PLC activity in presence of Cd. Result is presented as mean ± SD (n = 4). (**b**) Calcium imaging shows Cd induced elevation of intracellular Ca^2+^ level in mRTEC cells was eliminated by U73122, 2-APB, BAPTA and R-467. The mRTEC cells incubated with 1 μM Fluo-3 AM dye for 30 min, then pre-treated with U73122 (1 μM), 2-APB (50 μM), and BAPTA (10 μM) for another 30 min, then treated with Cd (5 μM) or Cd (5 μM) + R-467 (1 μM) for 1 hour and observed by Olympus IX73 microscopy. Scale bar: 20 μM. (**c**) Western blotting shows effects of U73122, 2-APB and BAPTA on Cd-induced apoptosis. Cells pretreated with U73122 (1 μM), 2-APB (50 μM), and BAPTA (10 μM) for 30 min, followed Cd treatment (5 μM) for 24 h, total proteins were extracted for western blotting analysis of expressions of cleaved caspase-3. (**d**) Effects of U73122, 2-APB and BAPTA on Cd-reduced cytotoxicity in mRTEC. Cells pretreated with U73122 (1 μM), 2-APB (50 μM), and BAPTA (10 μM) for 30 min, followed Cd treatment (5 μM) for 24 h, cell viability was evaluated by MTT assay. *Statistical significance between control and treatments, or Cd treatment and in presence of inhibitors, *P < 0.05, using Student’s t-test and one-way ANOVA followed by Duncan’s multiple range tests.

To confirm whether Cd induced elevation of intracellular Ca^2+^ level from Ca^2+^ store in ER through PLC-IP_3_ pathway in mRTEC cells, we measured PLC activity and intracellular Ca^2+^ levels with or without the inhibitors, including U73122 (inhibitor of PLC), 2-APB (inhibitor of IP_3_R), and BAPTA (intracellular Ca^2+^ chelator). Cd induced activation of PLC activity (Fig. 2a) and Cd induced intracellular Ca^2+^ level elevation in mRTEC cells was suppressed by the inhibitors, U73122 and 2-APB, BAPTA, and R-467 (Fig. 2b). The results were consistent with previous study that Cd induced elevation of intracellular Ca^2+^ level from Ca^2+^ store in ER through PLC-IP_3_ pathway. R-467 increased instead of decreased Cd-induced activation of PLC activity (Fig. 2a) but it could effectively restore intracellular Ca^2+^ level (Fig. 2b), the mechanism was further studied in the following sections. In addition, Cd induced intracellular Ca^2+^ elevation did not depend on CaSR, since it could not be blocked by CaSR antagonist NPS 2390 (Fig. 2b). Although block of PLC-IP_3_ pathway and chelation of intracellular Ca^2+^ inhibit Cd induced apoptosis (Fig. 2c), it did not prevent Cd to reduce cell viability (Fig. 2d), suggesting intracellular Ca^2+^ signaling was important for cell and might more complex mechanisms were involved.

### R-467 reversed Cd-altered p38 and ERK signaling pathways

**Figure 3:**
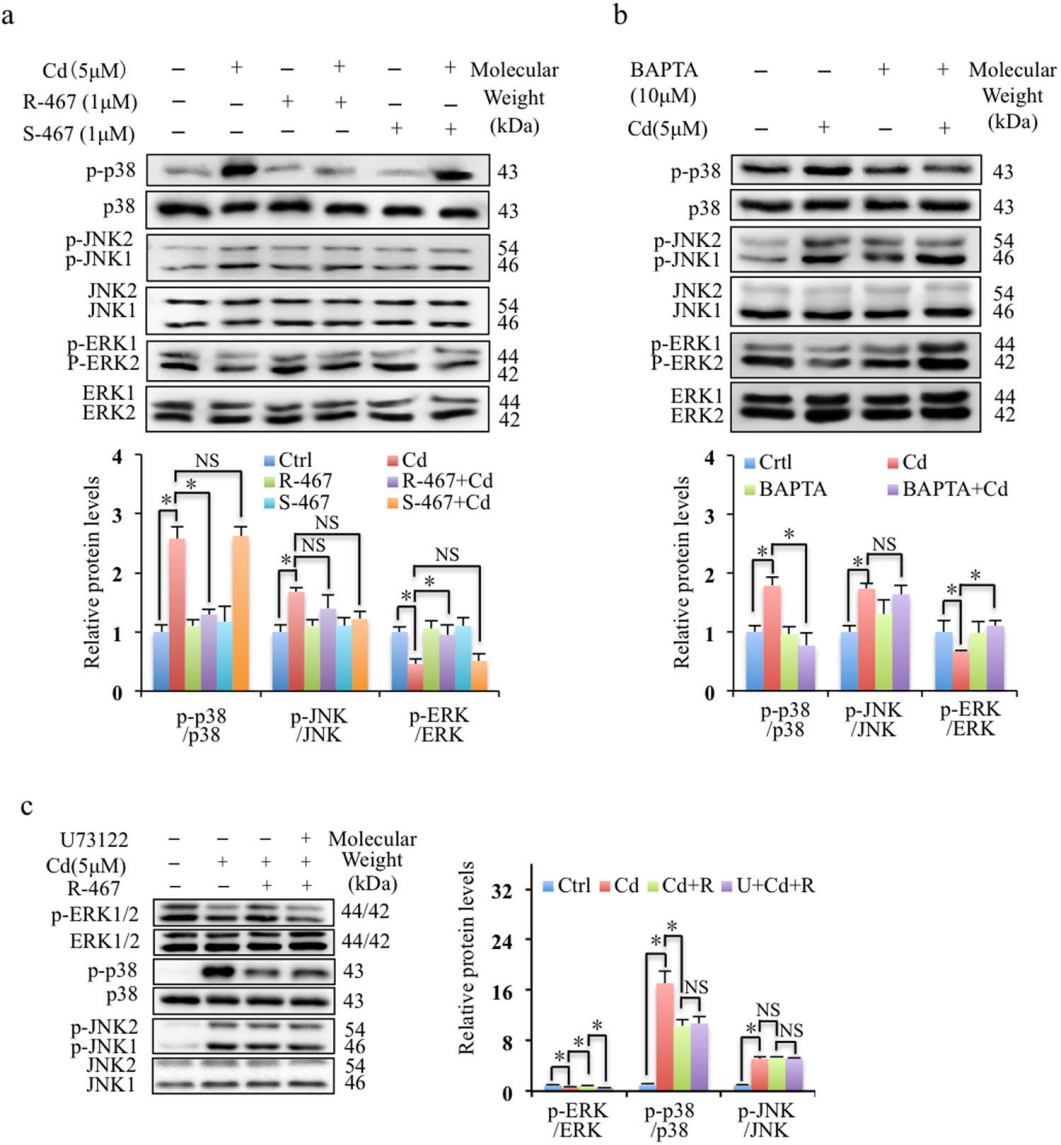
Effect of calcimimetics on Cd-regulated MAPK signaling pathway. (**a**,**b**) Effects of Cd, calcimimetics and intracellular Ca^2+^ chelator (BAPTA) on activation of MAPK signaling pathway. Cells treated with Cd treatment (5 μM), co-treated with calcimimetics R-467 and S-467 (1 μM), or pre-treated with BAPTA (10 μM) for 30 min, followed Cd treatment (5 μM) for 24 h, total proteins were extracted for western blotting analysis. Activations of and p38 MAPK and JNK represented by *in vitro* phosphorylations of p38 MAPK and JNK and showed significantly increase in response to Cd treatment. R-467 suppressed Cd-induced activation of p38 MAPK, but not JNK. Activation of ERK was decreased by Cd treatment, but it was increased when co-treated with R-467. BAPTA suppressed Cd-induced activation of p38 MAPK, but not JNK, and BAPTA increased Cd-suppressed activation of ERK. Total p38 MAPK, JNK and ERK1/2 served as loading control. (**c**) Inhibition of PLC on activation of MAPK pathway. Cells pretreated with U73122 (1 μM) for 30 min, followed by Cd treatment (5μM) or co-treated with R-467 (1 μM) for 24 h, total proteins were extracted for western blotting analysis of expression of phosphorylation of ERK, p38 and JNK. Total ERK1/2, p38 and JNK served as loading control. *Statistical significance between control and treatments or Cd treatment and in presence of inhibitors or calcimimetics, *P < 0.05, using Student’s t-test and one-way ANOVA followed by Duncan’s multiple range tests.

To further clarify the mechanism of R-467 reinstated Cd-induced intracellular Ca^2+^ level, we studied the activation of MAPK pathway. The results showed Cd induced activations of p38 and JNK while suppressed ERK1/2 MAPK signaling pathways (Fig. 3a). Effects of calcimimetics R-467 and S-467 on the activations of MAPK signaling pathway suggested that R-467 prevented Cd-induced activations of p38, but not JNK1/2 (Fig. 3a). In addition, Cd-suppressed activation of ERK1/2 was restored back by R-467 (Fig. 3a). Application of the intracellular Ca^2+^ chelator BAPTA indicated that Cd-induced elevation of intracellular Ca^2+^ level resulted in activation of p38, but not JNK1/2 (Fig. 3b). Interestingly, Cd suppressed activation of ERK1/2 was also restored back in the presence of BAPTA (Fig. 3b). It suggested that R-467 reinstated Cd-induced intracellular Ca^2+^ level to reverse Cd-altered p38 and ERK1/2 pathways. PLC inhibitor, U73122 eliminated the reverse effect of R-467 on Cd-suppressed activation of ERK, but did not alter R-467 reduced p38 activation (Fig. 3c). These results indicated that PLC activation was indispensable in reactivation of Cd-suppressed activation of ERK pathway.

### Cd-altered p38 and ERK signaling induced cytotoxicity

**Figure 4:**
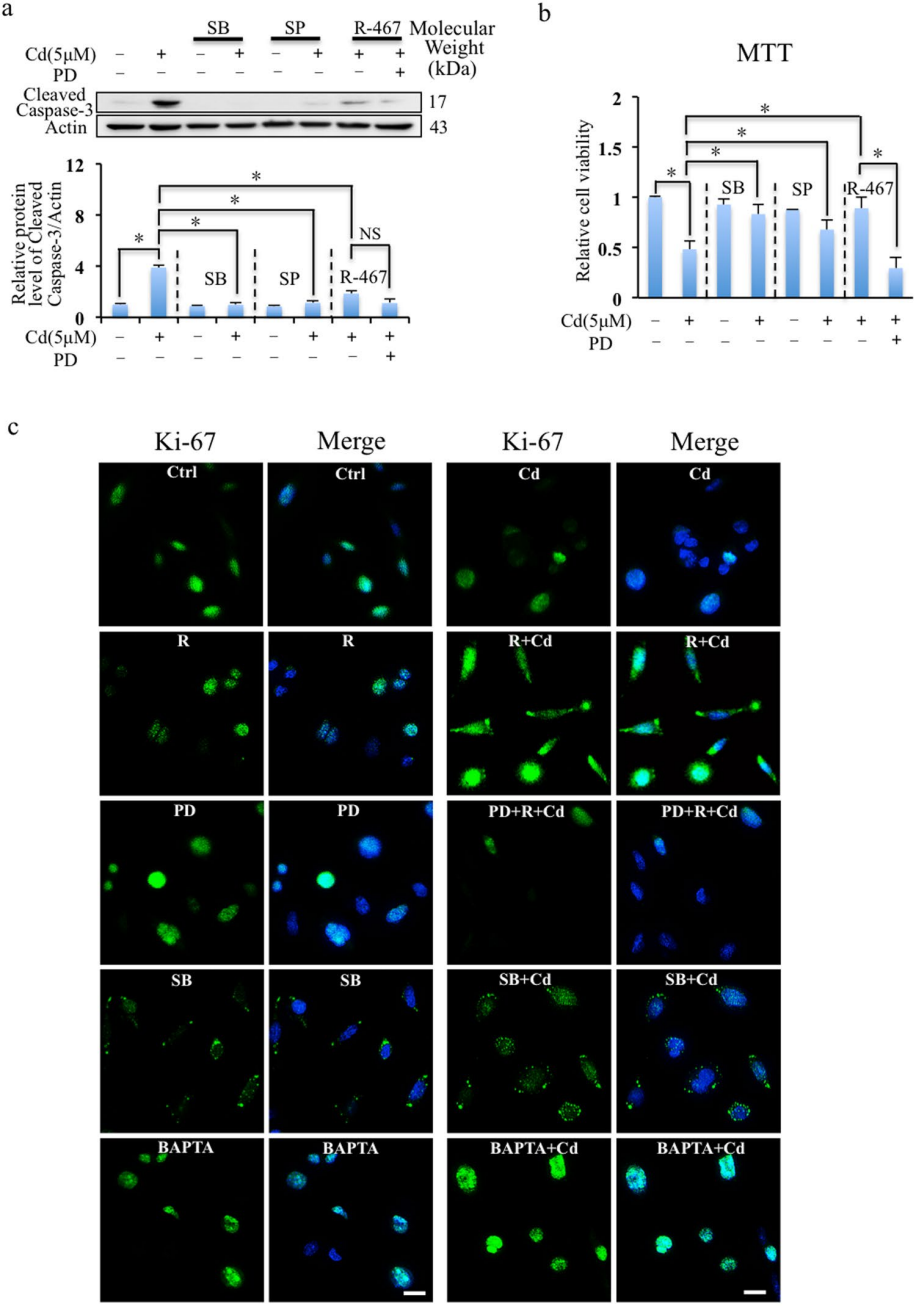
R-467 reduced apoptosis and induced cell proliferation. (**a**) Inhibition of MAPK signaling pathway on Cd and R-467-regulated apoptosis. Cells pretreated with SB202190 (10 μM), SP600125 (10 μM) or PD98059 (10 μM), for 30 min, followed by Cd treatment (5 μM) or co-treated with R-467 (1 μM) for 24 h, total proteins were extracted for Western blotting analysis of expression of cleaved caspase-3. (**b**) Inhibition of MAPK signaling pathway on Cd induced cytotoxicity. Cells pretreated with SB202190 (10 μM), SP600125 (10 μM) or PD98059 (10 μM), for 30 min, followed by Cd treatment (5 μM) or co-treated with R-467 (1 μM) for 24 h, the cell viability was evaluated by MTT assay. SB202190, SP600125 significantly inhibited Cd-induced cell death. Co-treatment of R-467 eliminated Cd-induced cytotoxicity, but suppressed by pretreatment of PD98059. Results are presented as mean ± SD (n = 4). *Statistical significance between control and treatments or Cd treatment and in presence of inhibitors or calcimimetics, *P < 0.05, using Student’s t-test and one-way ANOVA followed by Duncan’s multiple range tests. (**c**) Immunocytochemistry staining detection of Ki-67 expressions in mRTEC cells in Cd treatment (5 μM) or co-treated with R-467 (1 μM) for 24 h, or pretreat with SB202190 (10 μM), PD98059 (10 μM), or BAPTA (10 μM) for 30 min. The cells were counterstained with Ki-67 (green) and DAPI fluorescence (blue). Scale bar: 20 μM.

To investigate the roles of MAPK signaling pathway (i.e. p38 MAPK, JNK and ERK) in Cd-induced cell death, the specific inhibitors of these pathways, i.e. SB202190 (p38 inhibitor), SP600125 (JNK inhibitor) and PD98059 (ERK inhibitor) were applied. Inhibition of p38 and JNK reduced the expressions of cleaved caspase-3 (Fig. 4a) and decreased Cd induced cytotoxicity (Fig. 4b). It indicated that p38 and JNK pathways mediated Cd-induced apoptosis. In addition, ERK inhibitor, PD98059 did not alter R-467 reduced expression of cleaved caspase-3 in presence of Cd (Fig. 4a), which indicated R-467 could still prevent Cd-induced apoptosis when ERK activation was inhibited. However, PD98059 aggravated Cd-induced cytotoxicity in presence of Cd and R-467 (Fig. 4b), suggesting R-467-induced ERK activation played other roles in mRTEC cells.

To further understand the function of R-467 altered MAPK pathway, we detected expression and localization of Ki-67, which is used as a marker to indicate cell proliferation^51–53^. As shown in Fig. 4c, in control, most of the Ki-67 staining was completely superimposition with nucleus, and some of them localized to the periphery of the nucleus those are in proliferation. Cd suppressed expression of Ki-67 and restricted their localization in the nuclei (Fig. 4c), suggesting Cd inhibited cell proliferation. Co-treatment of Cd and R-467 not only significantly increased expression of Ki-67, but also improved translocalization of Ki-67 out of the nucleus (Fig. 4c), suggesting more cell proliferation. However, co-treatment of Cd and R-467-increased expression and translocalization of Ki-67 were suppressed by ERK inhibitor, PD98059 (Fig. 4c). It suggested co-treatment of Cd and R-467 induced expression of Ki-67 through ERK activation, which was necessary for translocalization of Ki-67 but was suppressed by Cd. In addition, enhanced p38 activation restricted translocalization of Ki-67 and SB202190 pre-treatment (p38 inhibitor) increased Ki-67 expression and stimulated most of the Ki-67 translocated to the periphery or out of the nuclei in presence of Cd (Fig. 4c), suggesting cell proliferation. Interestingly, BAPTA treatment increased Ki-67 expression but restricted localization of Ki-67 in the nuclei (Fig. 4c), suggested intracellular Ca^2+^ was necessary for translocalization of Ki-67 and cell proliferation. In hence, pretreatment of BAPTA could not increase cell proliferation, although enhanced ERK activation and suppressed p38 activation in presence of Cd (Fig. 3b). Taken together, these results indicated that increased ERK activation and suppressed p38 activation but without dysregulation of intracellular Ca^2+^ homeostasis could stimulate cell proliferation when exposed to Cd, which were coincidently satisfied when co-treatment with R-467.

### R-467 reduced Cd-evoked ROS generation, autophagic flux inhibition and apoptosis

**Figure 5:**
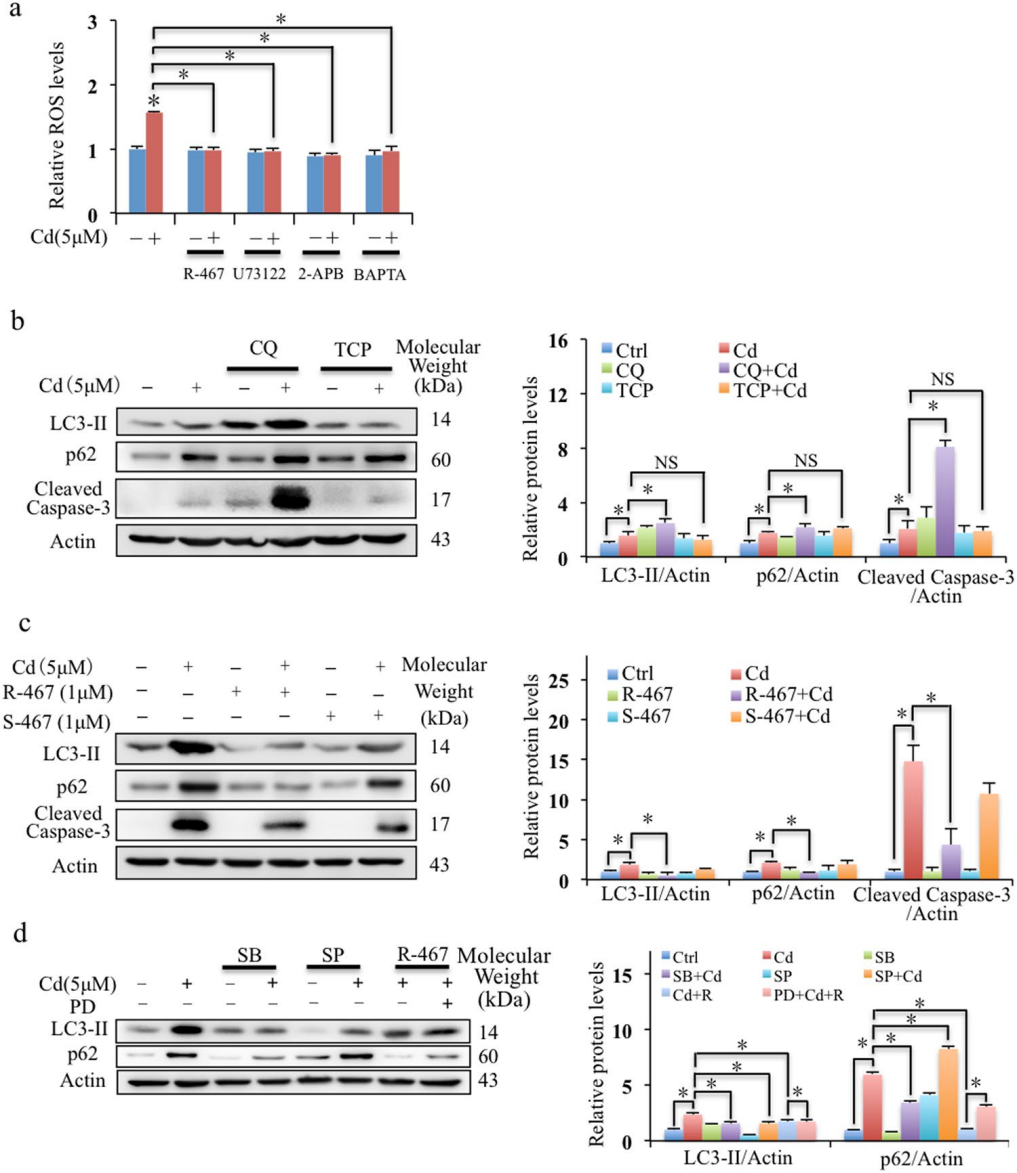
Effects of calcimimetics on Cd-induced ROS generation, autophagic flux inhibition and apoptosis. (**a**) Effect of U73122, 2-APB, BAPTA and Cd increased ROS generation in mRTEC cells. Cells pretreated with U73122 (1 μM), 2-APB (50 μM), and BAPTA (10 μM) for 30 min, followed Cd treatment (5 μM) or Cd (5 μM) + R-467 (1 μM) for 6 h, relative ROS levels in mRTEC cells were determined. Results are presented as mean ± SD (n = 4). (**b**,**c**) Western blotting shows effects of TCP, CQ and co-treatments of calcimimetics R-467 or S-467 with Cd on Cd-induced autophagy flux inhibition and apoptosis. Cells pretreated with TCP (100 μM) or CQ (20 μM) for 30 min, followed Cd treatment (5 μM) or Cd (5 μM) + R-467 (1 μM) for 24 h, total proteins were extracted for western blotting analysis of expressions of LC3-II and p62, and cleaved caspase-3. (**d**) Inhibition of MAPK signaling pathway on Cd and R-467-regulated cell autophagy. Cells pretreated with SB202190 (10 μM), SP600125 (10 μM) or PD98059 (10 μM), for 30 min, followed by Cd treatment (5 μM) or co-treated with R-467 (1 μM) for 24 h, total proteins were extracted for Western blotting analysis of expression of LC3-II and p62. *Statistical significance between control and treatments or Cd treatment and in presence of inhibitors or calcimimetics, *P < 0.05, using Student’s t-test and one-way ANOVA followed by Duncan’s multiple range tests.

To understand the role of Cd-increase intracellular Ca^2+^, ROS generation in mRTECs was determined. Cd significantly evoked generation of ROS in mRTEC cells (Fig. S2a), which could be abolished by inhibitors, U73122 and 2-APB, and intracellular Ca^2+^ chelator, BAPTA (Fig. 5a). This suggested Cd increased ROS generation by elevation of intracellular Ca^2+^ level and depended on PLC-IP_3_ pathway. To determine the roles of ROS generated by Cd exposure in Cd induced mRTEC cell death, TCP (ROS scavenger) and CQ (autophagy inhibitor) were applied. In Fig. S2b and Fig. S2c, it showed these two reagents reduced Cd-induced ROS generation but aggravated Cd-induced cell death. The rate of autophagic flux can be approximated by the amount of LC3-II with degradation of p62, whereas both of them are elevated when autophagic flux was impaired^6^. Cd increased the expressions of LC3-II and p62, which was aggravated by CQ (Fig. 5b). Addition of CQ could increase Cd-induced expression of cleaved caspase-3 (apoptosis marker) (Fig. 5b) and aggravate Cd induced cytotoxicity (Fig. S2b). These results indicated that Cd impaired autophagic flux, and increased apoptotic cell death. The pretreatment of TCP alleviated the Cd-induced accumulation of LC3-II and p62 (Fig. 5b), suggesting ROS could stimulate cell autophagy. However, scavenging of ROS by TCP aggravated instead of preventing Cd-induced cytotoxicity as show in Fig. S2b, suggesting that the cytotoxicity was not attributed to ROS generation and the induced autophagy signal under the exposure of Cd.

Co-treatment of R-467 eliminated Cd-induced generation of ROS (Fig. 5a), and R-467 effectively reducing Cd-increased of p62 accumulation and cleaved caspase-3 expression (Fig. 5c). This indicated that R-467 restored Cd-impaired autophagic flux and decreased Cd induced cell apoptosis, but which was not dependent on ROS generation. On the other hand, inhibition of p38 reduced the expressions of p62 but inhibition of JNK increased Cd-increased expression of p62 (Fig. 5d), indicating p38 pathway mediated Cd-impaired autophagic flux, whereas inhibition of JNK aggravated Cd-impaired autophagic flux. ERK inhibitor, PD98059 alone or in presence of Cd enhanced expression of p62 (Fig. S2d), and it also reversed co-treatment of R-467-reduced expression of p62 (Fig. 5d). Together with the effects of Cd and R-467 on activation of MAPK (Fig. 3a), the results indicated that R-467 restored autophagic flux by reversing Cd-altered p38 MAPK and ERK activations.

## Discussion

The expression of CaSR in renal proximal tubules had been report in rat^47–49^. The renal expression of CaSR plays important roles in calciotropic signals responsible for Ca^2+^ homeostasis^54,55^. Altered CaSR expression and disruption of Ca^2+^ homeostasis had been shown to be associated with renal insufficiency^56,57^. Previous studies suggested that Cd disrupted intracellular Ca^2+^ homeostasis through reducing the influx of extracellular Ca^2+^^23,24^, or increasing Ca^2+^ release from intracellular Ca^2+^ store^22,25^ and resulting in cell apoptosis of renal tubular cells^21,22^. In renal distal epithelial A6 cells, Cd disrupted intracellular Ca^2+^ homeostasis through a divalent cation receptor (CSR) mediated PLC-IP_3_ pathway^27^. Moreover, CSR was different from CaSR and CaSR agonist neomycin could diminish the effect of Cd on intracellular Ca^2+^^27^. However, the underlying mechanism and function of activation of CaSR on Cd-induced disruption of intracellular Ca^2+^ homeostasis and Cd-regulated pathways were still unclear. Interestingly, our previous study in gill cells of Japanese eels indicated that activation of CaSR and it mediated PLC-ERK pathway could as a protective pathway to reduce Ca^2+^-induced cytotoxicity^50^. It naturally raised a question that whether there is crosstalk between CaSR and CSR for competition of PLC pathway. In present study, we investigated whether activation of CaSR affected Cd-induced cell death of renal proximal tubular cells. Calcimimetics as allosteric modulator of CaSR is currently being tested for the treatment of primary hyperparathyroidism, and CaSR-based therapeutics will likely be applicable to other disorders in which CaSRs are under- or overactive^58^. In this study, the R-enantiomer (R-467) was used, which is classified as a type II calcimimetic and functions as a positive allosteric modulator of CaSR to amplify receptor sensitivity to Ca^2+^ or other full agonists^59^. The S-enantiomer S-467 is known to be less active^60^. Activation of CaSR by R-467 effectively protected renal cells from Cd-induced cytotoxicity and reducing Cd-evoked disruption of intracellular Ca^2+^ homeostasis, followed ROS generation, autophagic flux inhibition and apoptosis. The present study demonstrated that activation of CaSR could restore of the intracellular Ca^2+^ homeostasis and related physiological disorders.

Previous studies had show that Cd disrupted intracellular Ca^2+^ homeostasis, resulting in cell apoptosis in a variety of cells^9,16–19^, including renal tubular cells^21,22^. One source of disruption of cadmium on intracellular Ca^2+^ homeostasis was through increasing Ca^2+^ release from ER store mediated by PLC-IP_3_ pathway^18^. Our data confirmed this notion by pretreatment of U73122 and 2-APB, inhibitors of PLC and IP_3_ receptor respectively, inhibited Cd-induced elevation of intracellular Ca^2+^ levels in mRTEC. However, inhibition of PLC and IP_3_ receptor by U73122 and 2-APB, and chelation of intracellular Ca^2+^ by BAPTA could not inhibited Cd-induced cytotoxicity of mRTEC. It indicated that there should be other pathways mediated Cd-induced cytotoxicity and the underlying mechanism is more complex than our speculation. On the other hand, our results suggested that activation of CaSR by R-467 reduced Cd-evoked disruption of intracellular Ca^2+^ homeostasis in mRTECs, which was similar with the effect of CaSR agonist neomycin on Cd-increased intracellular Ca^2+^ in renal distal epithelial A6 cells^27^. The co-treatment of Cd + R-467 increased even higher PLC activity than Cd, but they displayed reverse effects on intracellular Ca^2+^ level. However, what elements decide the downstream effectors and fate of PLC pathway needs further study.

Furthermore, previous study suggested Cd-induced intracellular Ca^2+^ elevation resulted in induction of ROS, triggering cell apoptosis^19,61^. Pretreatment with BAPTA attenuated Cd-induced ROS in the neuronal cells^19,61^. In this study, our results confirmed that Cd induced elevation of intracellular Ca^2+^ level resulted in ROS generation in mRTEC. Notably, previous studies also had indicated that Cd induced autophagy through ROS-activated pathways^22,62^. Cd recruited Ca^2+^ and ROS to act as 2nd messengers to control key Ca^2+^- and redox-sensitive molecular switches dictating cell function and fate. Severe ROS/Ca^2+^ signals activate cell death, whereas low localized Ca^2+^ and ROS levels promote cellular adaptation and survival^63^. In addition, ROS promoted autophagic flux serves as a cell survival mechanism to protect cell death^64–66^. The data in present study showed Cd induced generation of ROS and intracellular ROS in mRTEC cells stimulated autophagy, but Cd impaired autophagic flux (accumulation of p62), which contributed to apoptotic cell death. It suggested Cd induced ROS generation and autophagy signal as passive responses of mRTEC cells to promote adaptation and cell survival in Cd exposure. Autophagy is considered as an adaptive and protective mean against Cd-induced damage and dysfunction. Hence, inhibition of autophagic flux, such as by CQ, aggravated Cd-induced apoptosis. More importantly, activation of CaSR by R-467 could eliminate Cd-induced generation of ROS, but restore Cd-impaired autophagic flux (reduced expression of p62) and reduce Cd-induced apoptosis (reduced expression of cleaved caspase-3).

To further understand the molecular mechanisms of Cd-induced cytotoxicity and the protective roles of action of R-467, we detected the role of MAPK pathway. Previous studies had indicated Cd regulated activation of MAPK pathway (i.e. JNK, p38 and ERK) and induced cell apoptosis^35,36,67,68^. The JNK pathway usually mediates stress response and apoptosis^69–71^, and p38 activation was indicated to be involved in suppressing autophagy but promoting apoptosis^72–75^. Conversely, ERK is activated to induce cell proliferation^76^ or promote cellular adaptation and survival^63^. Our results showed Cd disrupt autophagic flux by p38 activation, which was consent with previous studies. In addition, the results indicated Cd induced apoptotic cell death through activation of p38 MAPK and JNK pathways, and Cd inhibited cell proliferation through reducing activation of ERK. It agreed with the effects of Cd on activation of MAPK in the human non-small cell lung carcinoma cell line, CL3^36^, human osteosarcoma cell line, MG63^77^, and human renal proximal tubular cells, HK-2^12^. In rat pheochromocytoma (PC12), human neuroblastoma (SH-SY5Y) cell lines and primary murine neurons, intracellular Ca^2+^ signaling mediated Cd-induced neuronal apoptosis via induction of activation of MAPK^61^. In mesangial cells, Cd-stimulated Ca^2+^ release from the endoplasmic reticulum induced activation of ERK, which leaded to predominantly autophagic cell death and a minor level of apoptotic cell death and resulted in nephrotoxicity^78^. In present study, the results suggested that in mRTEC cells, Cd-induced activation of p38 MAPK depended on intracellular Ca^2+^ signaling, while Cd-induced activation of JNK was intracellular Ca^2+^ independent manner. Pretreatment with BAPTA attenuated Cd-reduced activation of ERK. It indicated that different MAPK families mediated Cd-induced intracellular Ca^2+^ signaling in different cell types. Our previous study had suggested CaSR mediated PLC-ERK pathway to regulate calcium transport in gill cells^50^. Here, the results indicated that activation of CaSR by R-467 induced activation of PLC-ERK pathway while reduced activation of p38 MAPK, which was similar with effects of BAPTA on activation of p38 MAPK and ERK. It was notable that although both R-467 and BAPTA could restore intracellular Ca^2+^ level, p38 and ERK activations, R-467 instead of BAPTA prevent Cd-induced cytotoxicity and proliferation inhibition in mRTEC cells, suggesting R-467 might play important role in restoring back of intracellular Ca^2+^ to ER store or induce other pathways to stimulate cell proliferation that need further studies. Through reducing activation of p38 MAPK and increasing activation of ERK pathway, R-467 restored autophagic flux and reduced apoptotic cell death and increased cell proliferation. The results here indicated that activation of CaSR by R-467 could switch Cd-activated PLC-Ca^2+^-p38 MAPK to activate PLC-ERK pathway. However, it cannot rule out other possible mechanisms, such as R-467 might also stimulate other receptors to prevent Cd-induced cytotoxicity or alter the binding affinity of Cd onto its receptor CSR, or there might be interaction or cross-talking between the receptors (CSR and CaSR) and their other downstream effectors which were altered by R-467, and how can R-467 switch Cd-activated PLC-Ca^2+^-p38 MAPK to activate PLC-ERK pathway and what key elements decide the fate of PLC activation are still unclear. Therefore, the clarification of other pathways and deep mechanisms behind the protective effect of R-467 need further studies.

**Figure 6:**
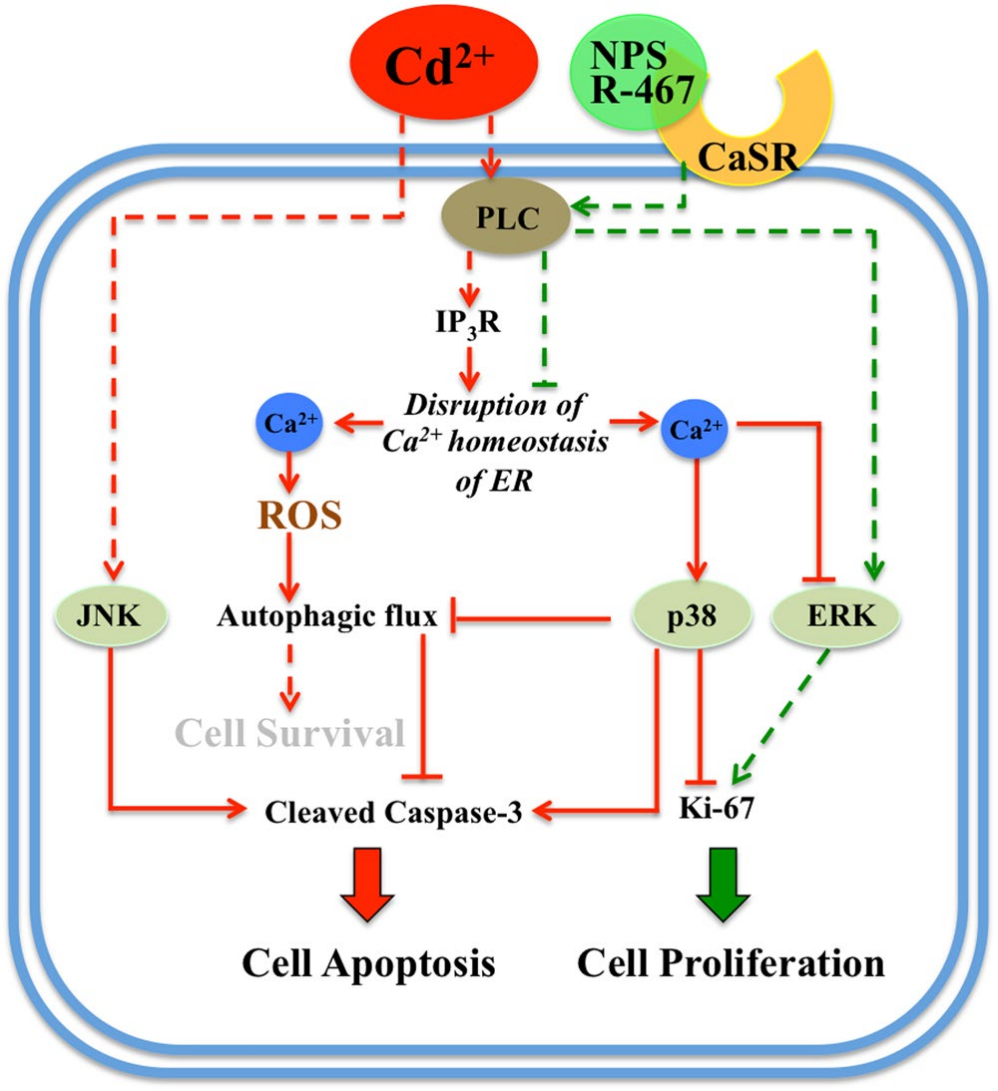
A schematic to illustrate calcimimetic R-467 reversed Cd-induced signaling pathways, followed reinstated intracellular Ca^2+^ level and ROS generation, restored autophagic flux, alleviated cell apoptosis and stimulated cell proliferation.

In summary, the results of this study demonstrated that Cd induced Ca^2+^ release from ER store through PLC-IP_3_ pathway in mRTEC cells, and elevation of intracellular Ca^2+^ level leaded to ROS generation. Cd-induced elevation of intracellular Ca^2+^ also leaded to p38 activation, which impaired autophagic flux, stimulated apoptosis and suppressed cell proliferation. In addition, Cd induced apoptosis through JNK activation, and inhibited cell proliferation through suppression of ERK activation. Activation of CaSR by R-467 reinstated intracellular Ca^2+^ level and ROS generation, restored Cd-impaired autophagic flux and reduced Cd-induced apoptosis, and increased cell proliferation by competing with Cd for PLC to switch Cd activated p38 MAPK to R-467 activated PLC-ERK pathway (Fig. 6). The identification of the activation of CaSR-mediated protective pathway in mRTEC cells sheds light on a possible cellular protective mechanism against Cd-induced kidney injury.

## Materials and Methods

### Chemicals

Cadmium chloride (Sigma, St. Louis, MO, USA) was dissolved in sterile distilled water to prepare the stock solutions (0–50 mM), aliquoted and stored at room temperature. Fluo-3/AM, and 2’7’-dichlorodihydrofluorescein diacetate (H2DCFDA) were purchased from Thermo Fisher Scientific (Waltham, MA, USA); U73122, 2-Aminoethoxydiphenylborane (2-APB), 1, 2-bis (o-aminophenoxy) ethane-N, N, N’, N’-tetraacetic acid (BAPTA/AM), PD98059, SB202190, SP600125 were purchased from Tocris Bioscience (Ellisville, MO, USA); α-tocopherol (TCP) and chloroquine (CQ) were purchased from Sigma (St. Louis, MO, USA); Calcimimetics NPS R-467 or S-467 were from NPS Pharmaceuticals (Salt Lake, UT, USA).

### Cell culture and treatments

Mouse renal tubular epithelial cells (mRTECs) and human kidney proximal tubule (HK-2) cells obtained from American Tissue Culture Collection (ATCC, Manassas, VA, USA) were grown at 37 °C in RPMI 1640 medium (pH 7.4) supplemented with 10% heat-inactivated fetal bovine serum (FBS), 100 U/ml penicillin, and 100 mg/ml streptomycin (pH 7.4) in a humidified atmosphere containing 5% CO_2_. RPMI 1640 medium, FBS, penicillin, and streptomycin were purchased from Gibco (CA, USA). The mRTEC and HK-2 cells (at 50% confluence) were treated with CdCl_2_ (0–20 μM or 0–50 μM respectively) for indicated time periods. In some experiments, cells were either untreated or pre-treated with kinase inhibitors for 30 min, i.e. U73122 (1 μM), 2-APB (50 μM), BAPTA/AM (10 μM), PD98059 (10 μM), SB202190 (10 μM), SP600125 (10 μM), TCP (100 μM) and CQ (20 μM). After 24 hours, the culture mediums were collected for cytotoxicity analysis, and the cell viability was measured by MTT assay. In some experiments, cell lysates were collected for western blotting analysis.

### Measurement of cell growth and cytotoxicity assay

After treated for 24 h, the culture mediums were collected to measure the activity of lactate dehydrogenase (LDH) using the LDH Cytotoxicity Assay Kit (Cayman) according to the manufacturer’s instructions. The absorbance at 490 nm was detected using a Synergy H4 Hybrid Multi-Mode Microplate Reader (BioTek, USA). Cell growth was measured by the MTT (3-(4,5-dimethyl-2-thiazolyl)-2,5- diphenyl-2-H- tetrazolium bromide) assay. In brief, cells were incubated in 100 μL MTT solution (0.5 mg/ml in RPMI 1640 medium) in 96-well plate for 4-h before the end of incubation. The supernatant was then discarded, and 100 μL DMSO was added to dissolve the colored product (formazan). The absorbance was measured at 540 nm (690 nm as reference) using a Synergy H4 Hybrid Multi-Mode Microplate Reader (BioTek, USA).

### PC-PLC assay

The mRTEC cells were lysed in radioimmunoprecipitation assay (RIPA) buffer (50 mM Tris of pH 7.4, 150 mM NaCl, 1% NP40) at 1, 2, 5 min post-treatment with CdCl_2_ (5 μM), or 2 min post-treatment with R-467 (1 μM), and R-467 (1 μM) + CdCl_2_ (5 μM). After centrifugation at 13 000 g for 15 min at 4 °C, the supernatant was collected and the total protein concentration was determined by the Bradford method (Bio-Rad). The protein lysates (15 μg/sample) were analyzed for PC-PLC activity using an Amplex Red PC-PLC assay kit according to the manufacturer’s instruction (Molecular Probes, Eugene, OR, USA). The reactions were incubated in darkness at 37 °C for 1 h. The fluorescence was measured with a Synergy H4 Hybrid Multi-Mode Microplate Reader (BioTek, USA) with excitation at 530 nm and emission at 590 nm.

### Measurement of intracellular calcium

To visualize the effect of Cd on intracellular Ca^2+^ levels in renal cells, mRTEC cells were seeded at a density of 2 × 10^4^ cells/well in 24-well plates. Next day, the cells were loaded with 1 μM Fluo-3/AM in the RMPI 1640 medium (phenol red free) for 30 min at 37 °C in the dark. After dye loading, the cells were washed twice with the medium. Then, the cells were pre-treated with inhibitors for 30 min, i.e. U73122 (1 μM), 2-APB (50 μM), and BAPTA/AM (10 μM), then added CdCl_2_ (5 μM) or CdCl_2_ (5 μM) + R467 (1 μM) for 1 hour. Finally, calcium imaging was observed by Olympus IX73 microscopy (Japan).

### ROS detection

The ROS level was measured using H2DCFDA (Thermo Fisher Scientific). Briefly, mRTEC cells were seeded at a density of 5 × 10^3^ cells/well in a 96-well plate. The next day, cells were treated with CdCl_2_ (5 μM) for 0.5, 0.75, 1.5, 3 and 6 h, or incubated in the presence or absence of CdCl_2_ (5 μM) for 3 h with R-467 (1 μM), or pretreated with U73122 (1 μM), 2-APB (50 μM), BAPTA/AM (10 μM), TCP (100 μM) and CQ (20 μM) for 30 min, followed by incubation with H2DCFDA for 30 min. Fluorescent intensity was recorded by excitation at 485 nm and emission at 525 nm using a Synergy H4 Hybrid Multi-Mode Microplate Reader (BioTek, USA).

### Western blotting

The mRTEC cells were lysed in radioimmunoprecipitation assay (RIPA) buffer (50 mM Tris of pH 7.4, 150 mM NaCl, 1%NP40) at period time after treatments. After centrifugation at 13 000 g for 15 min at 4 °C, the supernatant was collected and the total protein concentration was determined by the Bradford method (Bio-Rad). The protein lysates containing 40 μg total cellular protein in RIPA buffer were subjected to electrophoresis on 8–12% polyacrylamide gels. The gels were then blotted onto PVDF membranes (PerkinElmer Life Sciences, Foster City, CA, USA). Western blotting was conducted using rabbit monoclonal antibodies against CaSR (D6D9V), phospho-p38 mitogen-activated protein kinases (Thr180/Tyr182) (p-p38 MAPK), phospho- extracellular signal-regulated kinase (Thr202/Tyr204) (pERK1/2), phospho-stress-activated protein kinase/c-Jun NH(2)-terminal kinase (Thr183/Tyr185) (p-SAPK/JNK) and total p38, ERK, JNK; light chain 3-II (LC3-II) and p62; cleaved caspase-3, followed by incubation with horseradish peroxidase-conjugated goat anti-rabbit antibody (1:4000). Specific bands were visualized using chemiluminescent reagent (Western-lightening Plus, PerkinElmer Life Sciences). The blots were then washed in PBST and re-probed with rabbit anti-β-actin (1:1000). All the antibodies were purchased from Cell Signaling Technology (Shanghai, China). The intensity of each band was measured by Image J software (National Institutes of Health, USA).

### Immunofluorescence

To detect the expression of Ki-67, the mRTEC cells were treated with CdCl_2_ (5 μM) with or without R-467 (1 μM), or S-467 (1 μM) for 24 h, or pre-treated with PD98059 (10 μM), SB202190 (10 μM) for 30 min. The cells were fixed for 30 min in 4% Formaldehyde (FA, Sigma-Aldrich). Then, the cells were permeabilized with 0.1% Triton X-100 (Sigma-Aldrich) in PBS for 20 min. After blocked with 3% normal goat serum, the cells were incubated with mouse anti-Ki-67 (1:100, Cell Signaling Technology) antibody overnight at 4 °C, followed by 1 h incubation with Alexa Fluor 488 goat anti-mouse IgG (1:200, Molecular Probes, Invitrogen). After washing twice with PBS, cell nucleus was stained by the DAPI (Invitrogen) for several minutes. The cells were washed in PBS for 10 min and the cells were mounted, then examined by Olympus IX73 microscopy (Japan). To detect the expression of CaSR (anti-CaSR, 1:40, Thermo fisher Scientific) on the membranes of HK-2 and mRTEC cells, the process was performed without the permeabilization step.

### Statistical analysis

Drug treatments were performed in triplicate in each experiment and every experiment was repeated at least three times. All data are represented as means ± s.e.m. Statistical significance was assessed with Student’s *t*-test or one-way analysis of variance (ANOVA) followed by Duncan’s multiple range tests. Groups were considered significantly different if *P < *0.05.

## Electronic supplementary material

Supplementary Information
