# Supplementary material for: Activation of Ca2+-sensing receptor as a protective pathway to reduce Cadmium-induced cytotoxicity in renal proximal tubular cells
Source: Sci Rep. 2018 Jan 18;8:1092. doi: 10.1038/s41598-018-19327-9 (PMC5773512; doi:10.1038/s41598-018-19327-9)
Supplement: Supplementary file 1 — Supplementary Information [file 41598_2018_19327_MOESM1_ESM.pdf]

Activation of Ca^{2+}-sensing receptor as a protective pathway to reduce Cadmium-induced cytotoxicity in renal proximal tubular cells

Jie Gu[#1], Shuya Dai[#1], Yanmin Liu[1], Haitao Liu[1], Yao Zhang[2], Xingqi Ji[1], Feng Yu[1], Yang Zhou[1], Liang Chen[1], William Ka Fai Tse[3], Chris Kong Chu Wong[4], Binghai Chen[5], Haifeng Shi[*1]

[1] Institute of Life Science, Jiangsu University, Zhenjiang, Jiangsu 212000, CHINA,

[2] Medical Section, The Third Affiliated Hospital of Jiangsu University, Zhenjiang, Jiangsu 212000, CHINA,

[3] Faculty of Agriculture, Kyushu University, Fukuoka, JAPAN,

[4] Department of Biology, Hong Kong Baptist University, Hong Kong SAR,

[5] Department of urology, Affiliated Hospital of Jiangsu University, Zhenjiang, Jiangsu, 212000, CHINA,

[#] These authors contributed equally to this work

[*]Correspondence and requests for materials should be addressed to H.S. (email:shihf@ujs.edu.cn)

Supplementary

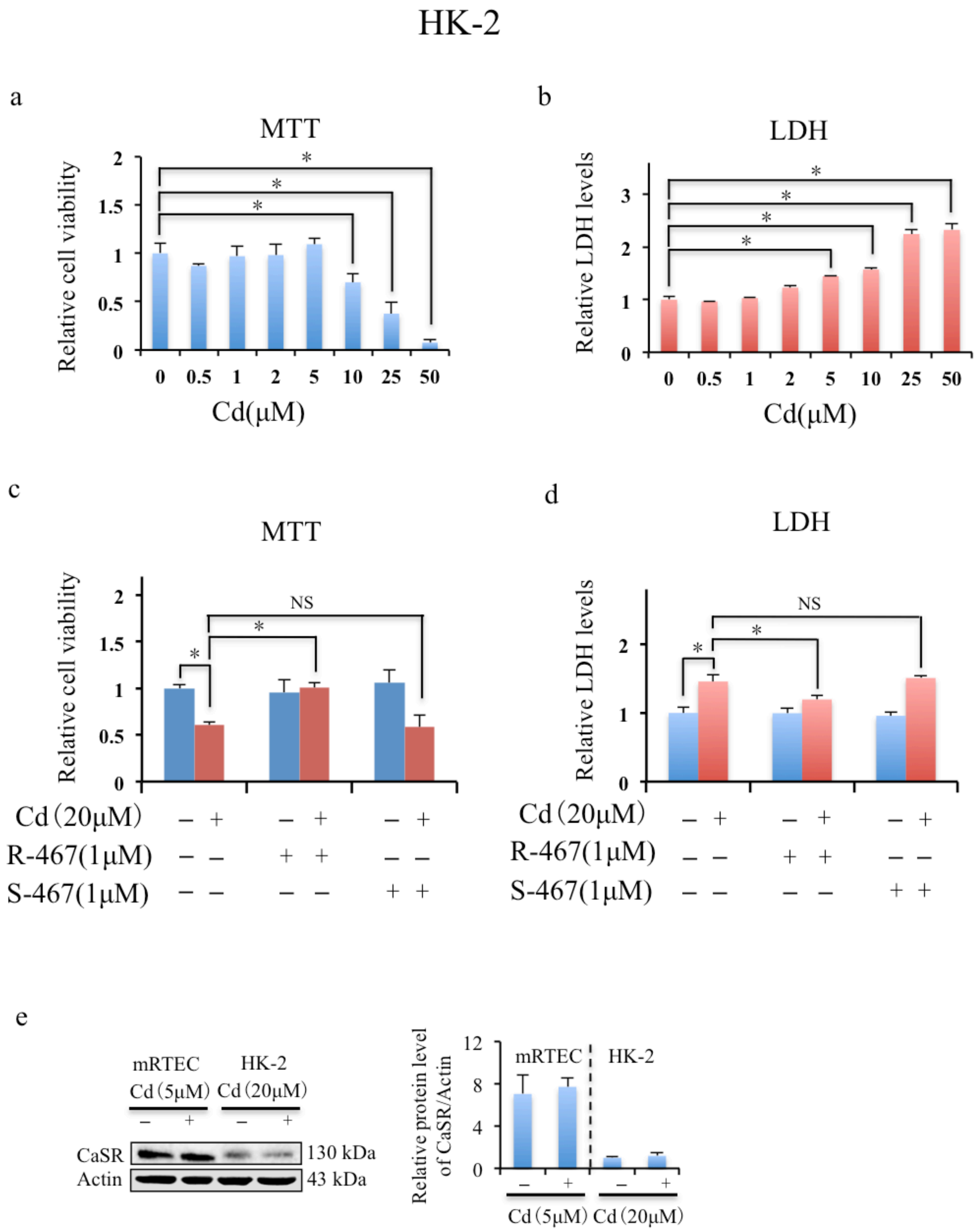

Fig.1. Cd induced cell death and cytotoxicity in HK-2 cells and effects of Cd on expression of CaSR in renal cells. (a, b) Cd induced cytotoxicity in HK-2 cells. After treated with 0-20

μM Cd for 24 h, cell viability of HK-2 cells was evaluated by MTT assay. The condition mediums were collected for LDH cytotoxicity assay. Results are presented as mean ± SD (n=4). (c, d) Effects of calcimimetics on Cd induced cytotoxicity in HK-2 cells. After treated with Cd (5 μM), R-467 (1 μM), S-467 (1 μM), Cd (5 μM)+R-467 (1 μM), or Cd (5 μM)+S-467 (1 μM) for 24 h, cell viability of HK-2 cells was evaluated by MTT assay. The condition mediums were collected for LDH cytotoxicity assay. Results are presented as mean ± SD (n=4). *Statistical significance between control and treatments, or Cd treatment and co-treatment of Cd+R-467, *$P<0.05$, using Student's t-test. (e) Western blotting shows effects of Cd on the expressions of CaSR in mRTEC and HK-2 cells.

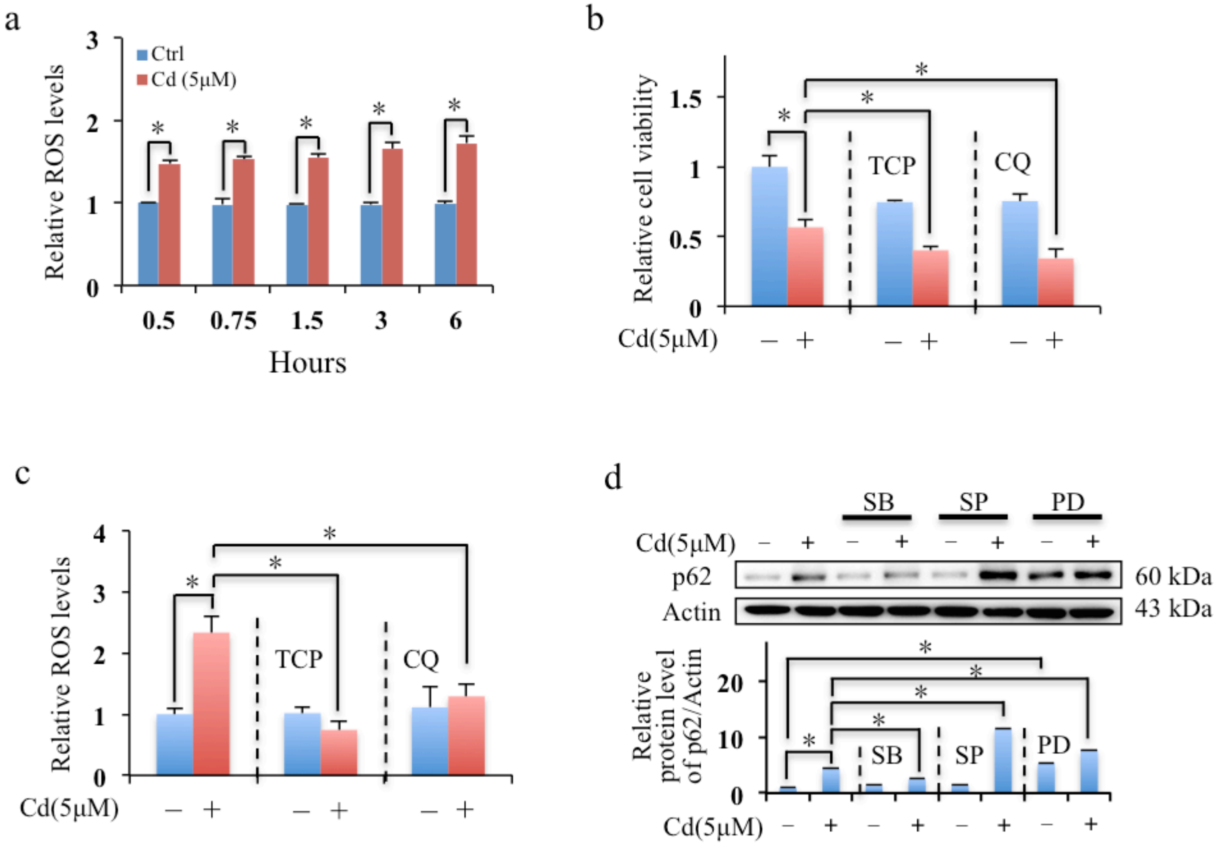

Fig.2. Effects of Cd on ROS generation, autophagic flux inhibition and apoptosis. (a) The relative ROS levels in mRTEC cells post-exposed to Cd (5 μM) for 0.5, 0.75, 1.5, 3 and 6 h. Cd slightly but significantly increased ROS generation in mRTEC cells. Results are presented as mean ± SD (n=4). (b, c) Effects of TCP and CQ on Cd induced cell death and ROS generation in mRTEC. Cells pretreated with TCP (100 μM) or CQ (20 μM), for 30 min, followed Cd treatment (5 μM) for 24 h, cell viability was evaluated by MTT assay; or followed Cd treatment (5 μM) and Cd (5 μM)+R-467 (1 μM) for 6 h, relative ROS levels in mRTEC cells were determined. TCP and CQ aggravated Cd-induce cell death, but suppressed Cd-induce ROS generation. Results are presented as mean ± SD (n=4). (d) Inhibition of MAPK signaling pathway on Cd-regulated p62 expression. Cells pretreated with SB202190 (10 μM), SP600125 (10 μM) or PD98059 (10 μM), for 30 min, followed by Cd treatment (5 μM) for 24 h, total proteins were

extracted for Western blotting analysis of expression of p62. *Statistical significance between control and treatments or Cd treatment and in presence of inhibitors or calcimimetics, * $P<0.05$, using Student's t-test and one-way ANOVA followed by Duncan's multiple range tests.
